# Can we use ChatGPT for Mental Health and Substance Use Education? Examining Its Quality and Potential Harms

**DOI:** 10.2196/51243

**Published:** 2023-11-30

**Authors:** Sophia Spallek, Louise Birrell, Stephanie Kershaw, Emma Krogh Devine, Louise Thornton

**Affiliations:** 1 The Matilda Centre for Research in Mental Health and Substance Use The University of Sydney Sydney Australia

**Keywords:** artificial intelligence, generative artificial intelligence, large language models, ChatGPT, medical education, health education, patient education handout, preventive health services, educational intervention, mental health, substance use

## Abstract

**Background:**

The use of generative artificial intelligence, more specifically large language models (LLMs), is proliferating, and as such, it is vital to consider both the value and potential harms of its use in medical education. Their efficiency in a variety of writing styles makes LLMs, such as ChatGPT, attractive for tailoring educational materials. However, this technology can feature biases and misinformation, which can be particularly harmful in medical education settings, such as mental health and substance use education. This viewpoint investigates if ChatGPT is sufficient for 2 common health education functions in the field of mental health and substance use: (1) answering users’ direct queries and (2) aiding in the development of quality consumer educational health materials.

**Objective:**

This viewpoint includes a case study to provide insight into the accessibility, biases, and quality of ChatGPT’s query responses and educational health materials. We aim to provide guidance for the general public and health educators wishing to utilize LLMs.

**Methods:**

We collected real world queries from 2 large-scale mental health and substance use portals and engineered a variety of prompts to use on GPT-4 Pro with the Bing BETA internet browsing plug-in. The outputs were evaluated with tools from the Sydney Health Literacy Lab to determine the accessibility, the adherence to Mindframe communication guidelines to identify biases, and author assessments on quality, including tailoring to audiences, duty of care disclaimers, and evidence-based internet references.

**Results:**

GPT-4’s outputs had good face validity, but upon detailed analysis were substandard in comparison to expert-developed materials. Without engineered prompting, the reading level, adherence to communication guidelines, and use of evidence-based websites were poor. Therefore, all outputs still required cautious human editing and oversight.

**Conclusions:**

GPT-4 is currently not reliable enough for direct-consumer queries, but educators and researchers can use it for creating educational materials with caution. Materials created with LLMs should disclose the use of generative artificial intelligence and be evaluated on their efficacy with the target audience.

## Introduction

### Background

Generative artificial intelligence (AI) large language models (LLMs) can now achieve high marks on medical competency exams [1], provide study plans for health students, and explain how a medication works. But can they provide truly accurate, quality health education? This viewpoint examined a popular LLM, ChatGPT, and used a case study to investigate if ChatGPT outputs meet the standards of educational health materials in the fields of mental health and substance use.

The incredible efficiency and human-like conversational tone of LLMs is an attractive feature for developing health educational materials. The traditional process involves tailoring materials to different audiences (eg, health workers, mental health and substance use clients, and parents), conducting literature reviews, consultations with experts, and editing text. Technological assistance with this time-consuming development process is worth investigating. However, ethical concerns and doubts about reliability or even inaccurate results that could be misleading may lead to hesitation toward using LLMs in this space.

To support this viewpoint, a case study was conducted on ChatGPT, an LLM with high-level skills in longer, organized text responses and varied writing styles. In addition, the popularity and widespread usage of ChatGPT makes this choice appropriate for this purpose, with a greater number of total visits than other LLM websites [2]. GPT-4 is the current version of ChatGPT and was developed by OpenAI. It is a general-purpose text generator and has been further trained with reinforcement learning to excel at generating conversational text [3].

### Potential Harms

We considered concerns regarding GPT-4’s accuracy and reliability, especially as the system does not provide any indication of its inner dialogue, such as reflections on the certainty of its claims. OpenAI is not transparent on how GPT-4 was trained, so it is unclear whether scientific research, often behind paywalls, was included in the vast data sets that were integrated into its network during training [3]. For example, a study into ChatGPT’s knowledge of clinical psychiatry found evidence for its promising accuracy, completeness, nuance, and speed, but also revealed a lack of pharmaceutical knowledge, which is typically found in textbooks rather than the web-based information ChatGPT was trained on [4].

Another concern regarding accuracy is so-called “hallucinations,” which occur when knowledge is missing from an LLM’s training set or when wrong connections are made in the probabilistic framework and the model unknowingly guesses, constructing an answer that sounds convincingly correct based on peripheral knowledge [5,6]. Schulman, co-founder of OpenAI, asserts that hallucinations have become less frequent as further iterations of ChatGPT are developed [3,7,8]. However, any occurrence of fabricated information in consumer medical education has the potential to cause harm, as users may not know to question GPT-4’s convincing outputs. A National Health Literacy survey in 2018 identified that only 11% of general population respondents strongly agreed that they could appraise the reliability of health information [9]. Educators are perhaps more likely to recognize falsehoods within their own area of expertise, but hallucinations remain a valid concern. Longer outputs, such as educational materials, have been found to be more likely to include hallucinations than shorter ones, as they typically include substantial text [7]. Professionals from other fields, such as law, have already demonstrated the pitfalls of accepting GPT-4’s output at face value without checking for hallucinations [10].

Methods to combat hallucinations are still developing. LLMs cannot identify when their knowledge is insufficient, making it difficult for developers to implement safeguards, such as hedging, where the output includes a cautionary note, such as “This is only probably correct.” As of June 2023, beta GPT-4 modes with internet browsing capacity might provide a potential safeguard against hallucinations, as they allow GPT-4 to fact-check and retrieve up-to-date sources, but this has not been thoroughly tested yet [7]. This feature could be beneficial or detrimental, as there is vast stigmatizing and untrue information on the internet regarding mental health and substance use. Some users may not question GPT-4’s sources as they are convincingly and authoritatively presented. It is therefore critical that educators and researchers approach seemingly factual output from LLMs through a critical lens [11,12]. Rather than repeatedly prompting GPT-4 until we identify a hallucination, our methodology focuses on the quality and use of evidence-based sources in GPT-4’s initial responses to prompts.

We also investigated if GPT-4’s outputs are accessible, unbiased, or included any potentially false or stigmatizing language. In relation to accessibility, the reading level of GPT-4’s outputs could restrict its use in consumer health education [13]. Comprehending written text is only one aspect of health literacy; in addition to this, modifying text for individuals’ accessibility and cultural context has been identified as an important aspect of improving health literacy [14]. With efficiency and tailored writing styles, GPT-4 has the potential to meet this need. However, if asked to write text for diverse audiences, outputs may contain possible cultural bias, gender bias, or other stigmatizing material due to a lack of representation of cultural sensitivities and diversity in the materials the model has been trained with. Already, a lack of training data from African countries has been identified as a limiting factor of LLMs [15]. The evidence supports that health education developed for and with specific demographics, such as youth or Aboriginal and Torres Strait Islander students, has higher acceptability and meets their needs [16]. We acknowledge the need for clinical, research, and lived-experience expertise, especially in relation to producing educational materials on complex health topics (eg, mental health and substance use) for minority populations for which LLMs may not be an appropriate tool.

Our viewpoint adds weight to the growing need for guidelines and instructions for the use of GPT-4 as an LLM in medical education [17-19]. The supporting case study specifically focused the use of GPT-4 in mental health and substance use education. It explored the strengths and limitations of GPT-4 in a variety of educational strategies and audiences, including the practical applications and ethical concerns of GPT-4 more broadly. Using authentic materials and varied prompts, we identified areas of concern and possible solutions. These materials included factsheets and real-world user queries from national educational prevention and harm reduction portals on which the authors have worked. Cracks in the Ice [20] and Positive Choices [21] are award-winning national translational web-based portals that have been accessed by over 1 million and 3 million website users, respectively, and have informed national and state-based policies [20-23]. These portals provide multimedia, evidence-based educational material to a variety of audiences, including parents, teachers, students, family, people who use substances, friends, and community leaders. Additionally, GPT-4’s responses to these prompts were scrutinized with the same tools that we use for the development of our own materials. Furthermore, we aimed to use our investigation into GPT-4’s quality to generate discussion about the use and evaluation of LLM-generated consumer materials.

## Methods

### Materials and Prompts

To maximize authenticity, we used real-world mental health and substance use materials in which the authors have project expertise. From the Cracks in the Ice and Positive Choices portals, materials were directly and indirectly utilized as prompts for GPT-4. This included simulated direct user queries submitted to GPT-4 and GPT-4–generated educational materials indirectly based on evidence-based factsheets.

#### Simulated Direct User Queries

Emails and user requests sent to the Positive Choices [21] and Cracks in the Ice [20] portals were reviewed by the authors; specifically, the latest 100 messages for Cracks in the Ice and the latest 200 messages for Positive Choices were included. Those related to technical issues (eg, I need help logging in), events (eg, how to access a webinar recording), and general questions about the portals themselves (eg, how to order booklets) were excluded. The remaining help-seeking and content-related queries were reviewed, deidentified (ie, removal or changing of names or locations), and summarized for brevity and confidentiality. We chose 5 queries from each portal that represented the widest variety of queries, and these were input into GPT-4 to recreate direct, user-to-AI queries from the general public. Grammatical errors were left in place to capture authentic, real-world communication (Textbox 1).

**Examples of queries to Positive Choices (1) and Cracks in the Ice (2) used to prompt GPT-4.**

1. “I have an anxious 16 year old son who hasnt yet started drinking alcohol and i would like to be able to help him BEFORE he starts drinking alcohol”
2. “I curious about methamphetamine use combined with anabolic steroids. I know a few people that are bodybuilding and also use rec methamphetamine (ice). Any info would be appreciated”

#### Evidence-Based Factsheets

Expert project coordinators from the Positive Choices and Cracks in the Ice portals selected multiple factsheets for various audiences (ie, youth, teachers, parents, people who use substances, and health professionals) and various substances to reflect the breath of available resources. A primary author unconnected to the portals then conducted a second review of the selected factsheets and made a final selection of 4 factsheets, 2 from each portal (Multimedia Appendix 1). These final 4 factsheets were selected to cover a number of different target audiences and represent a range of educational topics, as seen in Table 1.

These materials were used directly and indirectly in prompts that recreated educators’ usage of GPT-4. We anticipated that educators may use GPT-4 to draft and edit text and designed prompt templates that account for these 2 strategies. This included indirectly referring to the factsheets’ topic when requesting educational materials from scratch and directly providing the selected factsheets’ text to GPT-4 and requesting edits. To investigate how the quality of GPT-4 outputs varies with prompt engineering, we also investigated simplistic and engineered prompts. The engineered prompt structure featured a role, task, requirements, and instructions and was developed based on GPT-4’s internal prompts, university guides, and developer guides [24,25]. These prompts were also written to reflect best-practice communication guidelines in relation to mental health and substance use [26-28]. For each of the 4 factsheets, 3 prompts were applied in 3 steps within the same chat (Textbox 2), and a new chat was opened for each topic.

**Table 1:** The 4 factsheets chosen from the educational portals. The factsheets are subject to change, with these latest versions accessed in July 2023. The complete factsheets are provided in Multimedia Appendix 1.

Factsheet	Audience	Portal
Crystal methamphetamine use during pregnancy	Pregnant people who use ice	Cracks in the Ice
What are co-occurring conditions (‘comorbidity’)?	Health workers	Cracks in the Ice
How to help someone who has taken a drug	Students, teachers, and parents	Positive Choices
Drugs A-Z factsheet on cannabis	Teachers	Positive Choices

**Example template used when prompting GPT-4 with factsheet materials.**

Step 1: “Please write a 2-page factsheet about *Insert title of pre-existing factsheet*”.

Step 2: “Act as though you are an educator and please write a 2-page factsheet about *Insert title of pre-existing factsheet*. The factsheet’s target audience is Australian *target audience*. The factsheet should have a grade 8 readability level and supportive tone. Use these guidelines while writing: use person-first language, non-stigmatizing language, reducing harm, provide evidence based information, promote help-seeking behaviour, promote protective and preventative measures, strengths-based approach, reflect people’s lived experiences, avoid sensationalizing.”

Step 3: “Please edit this factsheet *Insert title of pre-existing factsheet*. The factsheet’s target audience is Australian *target audience*. The factsheet should have a grade 8 readability level and supportive tone. Use these guidelines while writing: use person-first language, non-stigmatizing language, reducing harm, provide evidence based information, promote help-seeking behaviour, promote protective and preventative measures, strengths-based approach, reflect people’s lived experiences, avoid sensationalizing. *Insert full text of expert created factsheet*”

### GPT-4 Protocol

The outputs analyzed in this viewpoint were generated on June 7 and 8, 2023, using GPT-4 Pro with the plug-in to browse with Bing BETA. The current version of GPT-4 received its last training data in September 2021. Prompts and outputs can be found in Multimedia Appendix 2.

User behaviour studies indicate that 15% to 17% of Google users do not refine their keywords in a second search [29,30]. With 93% of all internet searches conducted through Google, we assume that this lack of search refinement will carry over to GPT-4 [31]. Though health educators may conduct more follow-up prompting than direct users, a conscious decision was made not to conduct follow-up questions and requests for edits. This decision prioritizes the accuracy, safety, and ethics of GPT-4’s initial outputs. Therefore, the materials discussed were only prompted to GPT-4 once each.

### Evaluation Metrics

#### Sydney Health Literacy Lab

The Health Literacy Editor is a new tool recommended by the authors to refine the readability and accessibility of our consumer health education materials. The platform offers real-time insight into text, including readability grade, text complexity, passive voice, structure, and person-centered language. Most relevant to our investigation were the readability grade and text complexity, as we aimed to identify if GPT-4 can alter these aspects of its outputs based on different prompting. The recommended reading level of resources for the general public in Australia is grade 7 to 10 [13,32], and a lower percentage in the text complexity score is preferrable. To gain insight into the impact of prompting, the average grades and complexity scores of outputs for each type of prompt were calculated.

#### Communication Guidelines

Media guidelines for safe, respectful, and responsible communication about mental health and substance use are important to inform the development of nonstigmatizing health materials. Best-practice public communication guidelines for mental health and substance use were used to evaluate GPT-4–generated responses and consumer materials [26-28].

We selected the following 9 key guidelines from these resources: stigma reduction, promoting help-seeking behavior, minimizing harm, reflecting people’s lived experiences, avoiding sensationalizing, evidence-based, protective or preventative measures, person-centered language, and strengths-based or empowering language [27]. We evaluated how many of these 9 guidelines were used by each GPT-4 output and whether prompt engineering impacted this. Results representing a higher percentage of guidelines followed were desirable.

We also note the importance of providing referrals to professional support when discussing mental health and substance use. Therefore, we noted whether GPT-4 outputs included a disclaimer or referral.

#### Quality of Advice Provided

The authors evaluated the quality and accuracy of GPT-4’s application of mental health and substance use knowledge in an educational context. Any inaccurate information or hallucinations were noted. GPT-4’s ability to access, write, and provide evidence-based internet resources was also evaluated. We also considered GPT-4’s ability to tailor the communication of this information to the target audience.

### Ethical Considerations

To maximize authenticity, we used real-world mental health and substance use materials in which the authors have project expertise. From the Cracks in the Ice and Positive Choices portals, materials were directly and indirectly utilized as prompts for GPT-4. This included simulated direct user queries submitted to GPT-4 and GPT-4–generated educational materials indirectly based on evidence-based factsheets. No participant data on human subjects was collected for this opinion piece and case study. De-identified queries to public websites were used for illustrative purposes only, and no individual or identifying information were collected or reported.

## Results

### Summary of GPT-4 Outputs

A total of 22 queries or prompts were provided to GPT-4 as a part of this investigation. Of these, 12 were iterations of the 4 factsheets and the remaining 10 were direct user queries to educational and prevention portals. The results indicated how each type of prompts’ outputs adhered to the evaluation metrics. These results are reported as trends, as this case study’s small sample was not used in a statistical analysis within this viewpoint. Table 2 provides the average readability, text complexity, and adherence to the communication guidelines of outputs as well as the proportion of GPT-4 outputs that contained duty of care disclaimers or referrals.

**Table 2:** Results from the analysis of GPT-4 outputs with evaluation metrics. In total, 22 outputs were evaluated, including simulated direct user queries (n=10) and prompts for factsheets (n=12).

Metric	Direct user queries (n= 10)	Simple prompt to create factsheet from scratch (n=4)	Engineered prompt to create factsheet from scratch (n=4)	Engineered prompt for editing experts’ factsheet (n=4)	Original factsheets produced by experts (n=4)
SHeLL^a^ readability grade, mean (SD)	13.9 (1.52)	14.1 (1.74)	13.1 (3.20)	12.9 (1.20)	12.2 (1.44)
SHeLL text complexity (%), mean (SD)	24 (9.26)	33 (6.75)	23 (5.73)	27 (4.01)	27 (3.82)
Adherence to MH^b^ and AOD^c^ communication guidelines (%), mean (SD)	50 (1.75)	31 (1.80)	78 (1.22)	86 (1.64)	89 (0.71)
Duty of care: disclaimer or referral to professional, n (%)	6 (60)	2 (50)	4, (100)	3 (75)	3 (75)

^a^SHeLL: Sydney Health Literacy Lab.

^b^MH: mental health.

^c^AOD: alcohol and other drugs.

### Evaluation Metrics

#### Sydney Health Literacy Lab

Although no GPT-4 outputs nor original, expert-produced factsheets met the guidelines of a grade 7 to 10 readability level [13,32], the mean SHeLL readability grade improved with prompt engineering and providing text to edit. The average grades of the outputs for direct user queries and simple prompts were higher at 13.9 (SD 1.52) and 14.1 (SD 1.74), respectively, compared to the outputs for the 2 types of engineered prompts at grades 13.1 (SD 3.20) and 12.9 (SD 1.20). The average lowest and most desirable reading level was achieved by expert-developed factsheets (grade 12.2, SD 1.44). In addition to readability, the Sydney Health Literacy Lab measured the text complexity of GPT-4 outputs and evidence-based factsheets. Both featured a desirably low text complexity rating, varying from 24% to 33%, indicating a low number of uncommon words, medical jargon, and acronyms.

#### Communication Guidelines

Engineered prompting requesting the use of specific communication guidelines resulted in greater adherence to these guidelines. GPT-4’s responses to direct user queries and simple prompting of factsheets featured lower average adherence to communication guidelines in comparison with responses to engineered prompts. The relevance of each guideline may have varied for the different topics addressed in the prompts, but one clear pattern was identified: all outputs, even those with simple prompts, featured person-centered language. This leads us to consider that person-centered language may be integrated into GPT-4’s training or filters. Despite the commitment to person-centered language, GPT-4 was not able to use other nonstigmatizing language consistently, with 23% (5/22) of the outputs analyzed featuring at least 1 stigmatizing phrase (Textbox 3).

Outputs in response to engineered prompts featured disclaimers and a cautionary tone more so than outputs in response to direct queries and simple prompts, as seen in Textbox 4.

**Examples of stigmatizing language in GPT-4’s outputs for a direct user query (1) and the production of a factsheet from a simple prompt (2).**

1. “Dealing with someone who may be *abusing* drugs and exhibiting violent behavior can be distressing and potentially dangerous.”
2. “Approach the person when they are *sober*…”

**Examples of GPT-4’s disclaimers and referrals in response to a direct user query (1), a simple prompted factsheet (2), and an engineered prompted factsheet (3).**

1. “If the situation continues and you are worried about the well-being of your neighbor, you might consider reaching out to local social services. They may be able to provide resources or interventions that can help.”
2. “Note: This factsheet provides a general overview. Each individual's situation may vary, and anyone struggling with meth use during pregnancy should seek help from a healthcare professional.”
3. “Please note: While this factsheet provides a brief overview of the topic, it is recommended that health workers seek further training and resources for a more in-depth understanding of co-occurring conditions, including ice use and mental health disorders.”

#### Quality of the Advice Provided

The outputs analyzed in this viewpoint featured a generally high level of accuracy and no hallucinations. This is a promising finding regarding the accuracy of GPT-4’s knowledge of mental health and substance use. However, our analysis found that while GPT-4 can write about any topic, it lacks the breadth and depth of expertise and lived experience that human educators have. For example, the expert-created factsheets for pregnant women who use methamphetamine also included logically relevant information about breastfeeding, while GPT-4’s response did not. Only with engineered prompting did GPT-4 provide content regarding the important behavioral, environmental, and social aspects of mental health and substance use. This may be a result of GPT-4’s training and the limited amount and variety of evidence referenced in GPT-4’s internet browsing. The total of 25 websites that GPT-4 referred to were greatly outnumbered by the 55 high-quality, evidence-based citations that the expert-produced factsheets were based upon (Table 3). More specifically, GPT-4 used web-browsing in 2 of 10 direct user queries. GPT-4 accessed 1 journal article, 3 evidence-based resources, and 2 lower quality sources (a news article and Wikipedia). Web-browsing was also utilized for 5 of 12 factsheet responses. Of the 19 links referenced in factsheets, GPT-4 was able to access 3 journal articles. An additional 12 links featured evidence-based information, such as government health organizations, clinic websites, and even the authors’ own educational portals (Cracks in the Ice and Positive Choices). The 4 remaining links featured less scientific rigor, including 2 Wikipedia pages, the Foundation for a Drug-Free World from the Church of Scientology, and an ABC narrative on rehabilitation.

Another aspect we looked for when assessing the quality of advice was GPT-4’s ability to convincingly tailor complex information to target audiences. Its most relatable language and relevant tone were found in responses to engineered prompting where the target audience was specified. Typical Australian vernacular, such as “mate” and the spelling of “mum”, were consistently applied (Textbox 5). Without prompting the target audience, GPT-4 assumed a US-centric context.

**Table 3:** Types of references provided by GPT-4 compared to those provided in expert-produced factsheets.

Metric	Direct user queries (n=6)	Simple prompt to create factsheet from scratch (n=9)	Engineered prompt to create factsheet from scratch (n=7)	Engineered prompt for editing experts’ factsheet (n=3)	Original factsheets produced by experts (n=55)
Journal articles referenced, n (%)	1 (17)	0 (0)	3 (43)	0 (0)	49 (89)
Other evidence-based websites referenced, n (%)	3 (50)	6 (67)	3 (43)	3 (100)	6 (11)
Lower quality websites referenced, n (%)	2 (33)	3 (33)	1 (14)	0 (0)	0 (0)

**Examples of GPT-4 tailoring text for Australian students, teachers and parents (1) and Australian pregnant people (2).**

1. “Supporting a *mate* who is using drugs can be tough. Make sure you also take care of yourself”
2. “Babies whose *mums* used crystal meth might have a tough time after they're born. They might be fussy, cry a lot, or have trouble eating.”

## Discussion

### Principal Results

The outputs generated by GPT-4 in relation to enquiries for advice and information regarding mental health and substance use had good face validity, appearing to be evidence-based and of high-quality. However, further analysis demonstrated that GPT-4’s initial outputs did not meet the common criteria used when researchers develop educational materials (ie, good readability and nonstigmatizing language). For example, GPT-4’s initial outputs did not consistently adhere to the readability levels and communication guidelines requested in engineered prompting. GPT-4 was able to tailor information to target audiences; however, a lack of training on certain subpopulations may limit its applicability to produce accurate and unbiased information for minority populations [15]. With internet browsing enabled, we were able to gain insight into which resources GPT-4 utilized to fill gaps in its knowledge. With only a few scientific journal articles accessed, the overall quality of the chosen sources and websites was lower and more limited in comparison to expert-curated evidence. GPT-4’s initial outputs were very impressive and partly usable, but still featured inaccessibility, occasionally contained stigmatizing language, and lacked a thorough evidence base. It should also be noted that GPT-4 adopts a confident tone and academic language to engender trust in its output.

### Future Opportunities

#### Direct User Queries

We do not recommend that the general public uses GPT-4 in its current state for direct, personal health questions. While the response may appear convincing at face value, the quality of advice will vary depending on the prompt used and materials underlying the response. Though we found that prompt engineering can improve the safety and reliability of the output, people do not historically refine their searches [30]. Another consideration in the use of LLMs for health education purposes is that privacy is not afforded to conversations with GPT-4 [12]. The founding company, OpenAI, has confirmed that AI trainers, the people responsible for the reinforcement learning part of training, can review conversations to improve the model. Users can delete their data but cannot remove their prompt history from the trainers’ access [33]. Therefore, safety, privacy concerns, accessibility, biases, quality of evidence, and the need for more disclaimers indicate that GPT-4 is not ready for direct use by consumers for mental health and substance use advice. By extension, health practitioners, educators, and mobile health intervention personnel should not refer users to GPT-4 for addressing queries.

An area for future research and development of LLMs for direct user health enquiries could involve the use of open-source LLMs. These can be run locally and privately and be trained by researchers themselves on specific data sets [34,35]. These models, such as Large Language Model Meta AI (LLaMA), are available for the public to use; are noncommercial, smaller, and customizable; and have transparency around training [8]. Concerns regarding evidence-based training data could also be addressed with this type of LLM, as they can be custom trained on one’s own materials. However, open source LLMs are less linguistically gifted and conversational than GPT-4. Both ChatGPT and developments in other LLMs should be monitored and re-evaluated to identify when the above concerns are addressed.

#### Use By Educators and Researchers

For educators, we consider the current level of risk in GPT-4’s outputs to be acceptable when used with caution. Primarily, our findings indicate that human oversight is necessary and that while GPT-4 may be a useful tool in creating consumer educational materials, outputs must be edited and reviewed by subject experts. Specific advice for current and future use of GPT-4 when creating educational materials is provided in Table 4.

We also advise that educators disclose the use of any LLM when creating materials. By being transparent with audiences regarding how materials were developed, we can enable their use of health literacy skills and promote trust. When educators discuss their use of LLMs, they can bring attention to the nuances of this technology, particularly when it is cautiously wielded by experts. Already, a digital mental health intervention has used ChatGPT without informed consent, with some users believing they were communicating with a person [39]. Hopefully, this controversy has set an example to learn from rather than a new precedent.

**Table 4:** Advice for prompting GPT-4 and refining its outputs.

Consideration	How	Example
Prompt structure	Structure your prompt to explain the role GPT-4 should take on, the task you wish it to complete, the requirements of the task, and instructions on how to complete the task.	“Pretend you are a high school teacher (role) and create story about anxiety for your lesson (task). The story should include diverse characters and be 500 words long (requirements). When writing, use person-centered language and evidence-based information (instructions).”
Editing	Provide GPT-4 with a draft to edit, rather than requesting text from scratch. Our findings indicate this allows richer experience and evidence from human experts to shine through in the outputs.	“Edit the text below to shorten it to 500 words and make the tone engaging. *Insert draft*”
Target audience	Specify your target audience and location. GPT-4 automatically assumes that links, organizations, laws, and other advice should be relevant to the United States.	“Please write this for an audience of young mothers in rural Australia.”
Bias	When tailoring resources for minority groups, carefully review the output and refine it based on cultural sensitivity guidelines, including those with lived experience.	In mental health and substance use education, we reflect on communication guidelines [26] and co-design with lived-experience advisory boards [36].
Communication guidelines	Include communication guidelines and readability level in your initial prompt but expect to refine these. Our findings indicate GPT-4 will not adhere to all guidelines in its initial output, so continue prompting and conduct your own thorough edits.	“Please edit your previous output with a focus on lowering the readability level to grade 8.”
Evaluation	Evaluate GPT-4’s outputs thoroughly, using the most up-to-date metrics, measures, and guidelines of your field.	In mental health education, we would include data from the most recent National Study of Mental Health and Wellbeing statistics [37].
Plug-ins	Consider testing, evaluating, and utilizing a plug-in that enables GPT-4 to access scientific journals. Our investigation did not utilize or evaluate plug-ins, as this was outside of the scope.	Use plug-ins such as ScienceAI or Litmap [38].

### Limitations

GPT-4 and other generative AI models are rapidly developing, which places this study as a vital stepping stone to guide the future assessments of new iterations. In fact, we advise medical educators to continue to develop their strategies for AI usage as the technology develops. Starting with informal case studies in this viewpoint, we believe that trials of generative AI in health education will lead to further, evidence-based developments in safety and accuracy.

Not only will future versions of ChatGPT supersede GPT-4, but the current GPT-4 sits behind a paywall. The Pro version with Bing BETA internet browsing requires a paid subscription (US $20 per month). More broadly than this paper, we have concerns about the financial accessibility of LLMs and the subscription costs that enable access to accuracy-improving features. Consideration must also be given to people in low- and middle-income countries who are already at a disadvantage and are often unable to access the latest research due to journal paywalls [40], thus potentially compounding the cost of using GPT-4 at its best.

We also acknowledge that plug-ins are available to address some of the concerns we evaluated in this paper. In particular, access to scientific journals can be facilitated with plug-ins such as ScienceAI and Litmap [38]. However, the scope of this paper does not include the evaluation of various plug-ins, which are also rapidly developing. In addition, the primary aim of our evaluation was to evaluate GPT-4’s initial outputs to prompts without the assistance of prompt refining or plug-ins to be able to assess the baseline of its safety and accuracy.

### Conclusions

GPT-4 represents an exciting development for consumer medical education, however both direct users and educators should proceed with caution and disclosure. Our case study demonstrated, through a number of real-world examples, both the strengths and limitations of direct generative AI responses for mental health and substance use education for the general public. Our investigation indicates that GPT-4’s outputs are substandard to human experts, but has also led to the identification of a number of practical strategies that researchers and educators may follow to address accessibility, biases, and misinformation. We recommend that evaluations of the efficacy and acceptability of materials developed with LLMs are conducted in education and public health prevention efforts. This viewpoint is only a starting point, and we expect LLMs to continue developing rapidly. As OpenAI and other LLM providers tackle hallucinations and bias, ongoing evaluations of the safety and best practices of this technology will continue to be necessary, especially in the realm of medical education.

## Appendix

Multimedia Appendix 1 The 4 original factsheets developed by experts and used for prompting.

Multimedia Appendix 2 Transcripts of GPT-4 prompts and outputs.

## Abbreviations

AI: artificial intelligence
LLM: large language model
LLaMA: Large Language Model Meta AI
