# Supplementary material for: Can we use ChatGPT for Mental Health and Substance Use Education? Examining Its Quality and Potential Harms
Source: JMIR Med Educ. 2023 Nov 30;9:e51243. doi: 10.2196/51243 (PMC10722374; doi:10.2196/51243)
Supplement: Multimedia Appendix 1 [file mededu_v9i1e51243_app1.pdf]

CRYSTAL METHAMPHETAMINE USE DURING PREGNANCY

SUMMARY

- Using crystal methamphetamine ('ice') when you're pregnant can severely impact the health and development of your baby and put your own health at risk.
- There is no safe level of crystal methamphetamine use during pregnancy. Using the drug more frequently or in larger amounts increases the risk of health complications.
- If you think you might need help with crystal methamphetamine or other drug use, there is professional support available. You can ask your GP or obstetrician or call the National Alcohol and Other Drug Hotline on 1800 250 015 for a referral to a drug and alcohol service
- It's also important that you look after yourself and your baby in other ways during your pregnancy and after the birth. Your GP or obstetrician can give you advice on what to do and any additional supports you might need.

IS IT SAFE FOR ME TO USE CRYSTAL METHAMPHETAMINE WHILE I'M PREGNANT?

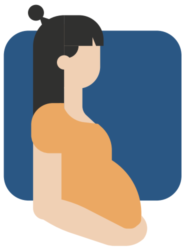

No. There is no level of crystal methamphetamine use that is safe for you and your baby when you're pregnant. The more often you use crystal methamphetamine during pregnancy, and the larger the amount of crystal methamphetamine you use, the greater your risk of health complications for you and your baby.

WHAT ARE THE EFFECTS OF CRYSTAL METHAMPHETAMINE USE DURING PREGNANCY?

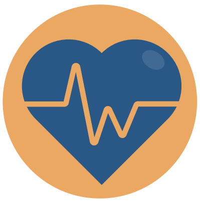

Crystal methamphetamine is a stimulant drug that speeds up your heart rate and puts strain on your body. This can increase your risk of health complications (such as brain, heart, liver, and kidney problems). These effects can also endanger your baby, increasing the risk of premature birth, miscarriage, or stillbirth.

Using crystal methamphetamine during pregnancy can also have other effects on your baby's health. For example, using crystal methamphetamine can make you feel less hungry, causing you to eat less. This can lead your baby to become malnourished and restrict their growth and development.

There is also some evidence that crystal methamphetamine use during pregnancy can impact a child over the long term. Studies have found associations between crystal methamphetamine use during pregnancy and poorer intellectual functioning, problem solving skills, short-term memory, and language development from birth to 16 years. There have also been associations with behavioural problems and learning difficulties. Babies may also be born small and/or premature, which increases their risk of needing intensive care and long-term health issues like diabetes, heart disease and lung problems.

Any drug use, including tobacco, alcohol, and illicit drug use during pregnancy can severely impact the health and development of your baby. There is no safe level of alcohol and other drug use during pregnancy.

IS IT SAFE FOR ME TO USE CRYSTAL METHAMPHETAMINE WHILE BREASTFEEDING?

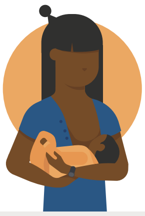

No. There is no level of crystal methamphetamine use that is safe during breastfeeding. This is because crystal methamphetamine can be released into breast milk and cause problems for your child's health and development. It's recommended that mothers don't breastfeed for at least 48 hours (2 days) after using crystal methamphetamine. Your GP or obstetrician can provide further advice on how to limit any risk to your baby.

I WANT TO STOP USING CRYSTAL METHAMPHETAMINE, BUT I NEED HELP. WHERE CAN I GO TO FOR SUPPORT?

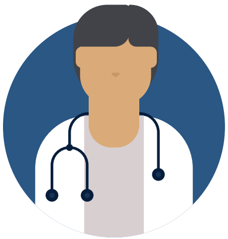

Several services throughout Australia can provide support to those experiencing problems with crystal methamphetamine and other drugs. Although it can be difficult to seek help, the sooner you reach out for support, the better.

Your GP or obstetrician can be a good starting point – they can discuss your concerns with you and provide referrals to other services that you might need. For free and confidential advice about alcohol and other drugs, you can also call the National Alcohol and Other Drug Hotline on 1800 250 015.

For links to more alcohol and other drug services in Australia, visit our **When and Where to Get Help page**.

WHAT ELSE CAN I DO TO LOOK AFTER MYSELF AND MY BABY THROUGH PREGNANCY?

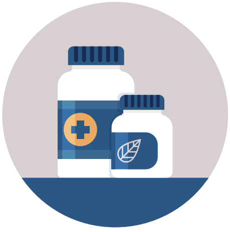

It's important that you look after yourself and your baby in other ways during your pregnancy and after the birth.

Prenatal and postnatal care appointments are vital for any pregnancy and will need to be attended regularly to ensure you are healthy and that your baby is safe and growing well.

Regardless of whether you stop using crystal methamphetamine or continue to use the drug during pregnancy, it's important that you inform your GP or obstetrician of your crystal methamphetamine use. They will be able to give you advice on any additional steps you may need to take to support the health of you and your baby. For example, you might need nutritional supplements if your appetite has been affected by your crystal methamphetamine use.

SOURCES:

1. Abar, B., LaGasse, L. L., Derauf, C., Newman, E., Shah, R., Smith, L. M., ... & Neal, C. (2013). Examining the relationships between prenatal methamphetamine exposure, early adversity, and child neurobehavioral disinhibition. Psychology of Addictive Behaviors, 27(3), 662.
2. Admon, L. K., Bart, G., Kozhimannil, K. B., Richardson, C. R., Dalton, V. K., & Winkelman, T. N. (2019). Amphetamine-and-opioid-affected births: incidence, outcomes, and costs, United States, 2004–2015. American journal of public health, 109(1),148-154.
3. Arria, A., Derauf, C., LaGasse, L., Grant, P., Shah, R., Smith, L., Haning, W., Huestis, M., Strauss, A., Grotta, S., Liu, J. and Lester, B. (2006). Methamphetamine and Other Substance Use During Pregnancy: Preliminary Estimates From the Infant Development, Environment, and Lifestyle (IDEAL) Study. Maternal and Child Health Journal, 10(3), pp.293-302.
4. Bartu, A., Dusci, L. J., & Ilett, K. F. (2009). Transfer of methylamphetamine and amphetamine into breast milk following recreational use of methylamphetamine. British journal of clinical pharmacology, 67(4), 455-459.
5. Brinker, M. J., Cohen, J. G., Sharrette, J. A., & Hall, T. A. (2019). Neurocognitive and neurodevelopmental impact of prenatal methamphetamine exposure: A comparison study of prenatally exposed children with nonexposed ADHD peers. Applied Neuropsychology: Child, 8(2), 132-139.
6. Chang, L., Smith, L. M., LoPresti, C., Yonekura, M. L., Kuo, J., Walot, I., & Ernst, T. (2004). Smaller subcortical volumes and cognitive deficits in children with prenatal methamphetamine exposure. Psychiatry Research: Neuroimaging, 132(2), 95-106.
7. Chang, L., Cloak, C., Jiang, C., Farnham, S., Tokeshi, B., Buchthal, S., Hedemark, B., Smith, L. & Ernst, T. (2009). Altered neurometabolites and motor integration in children exposed to methamphetamine in utero. NeuroImage, 48(2), pp.391-397.
8. Dean A., 2004, Psychostimulant use in pregnancy and lactation, Models of Intervention and Care for Psychostimulant Users 2nd Edition, Baker A., Lee N. and Jenner L. (eds), Commonwealth of Australia Monograph Series, 169-187, Australian Government Department of Health and Ageing, Canberra.
9. Della-Grotta, S., LaGasse, L., Arria, A., Derauf, C., Grant, P., Smith, L., Shah, R., Huestis, M., Liu, J. & Lester, B. (2009). Patterns of Methamphetamine Use During Pregnancy: Results from the Infant Development, Environment, and Lifestyle (IDEAL) Study. Maternal and Child Health Journal, 14(4), pp.519-527.
10. Jenner, L. and Lee, N. (2008). Treatment Approaches for Users of Methamphetamine: A Practical Guide for Frontline Workers. Australian Government Department of Health and Ageing, Canberra.
11. Kalaitzopoulos, D., Chatzistergiou, K., Amylidi, A., Kokkinidis, D. & Goulis, D. (2018). Effect of Methamphetamine Hydrochloride on Pregnancy Outcome. Journal of Addiction Medicine, 12(3), pp.220-226.
12. Kunkler, C., Lewis, A. J., & Almeida, R. (2022). Methamphetamine exposure during pregnancy: A meta-analysis of child developmental outcomes. Neuroscience and biobehavioral reviews, 138, 104714. **https://doi.org/10.1016/j.neubiorev.2022.104714**
13. Lee N, 2004, 'Risks associated with psychostimulant use' Models of Intervention and Care for Psychostimulant Users 2nd edition, Baker A, Lee N and Jenner L (eds), Commonwealth of Australia Monograph Series, 51-59, Australian Government Department of Health and Ageing, Canberra.

SOURCES:

14. Maya-Enero, S., Candel-Pau, J., Rebollo-Polo, M., Candela-Cantó, S., de la Torre, R. and López-Vílchez, M. (2018). Central nervous system malformation associated with methamphetamine abuse during pregnancy. Clinical Toxicology, pp.1-3.
15. Nguyen, D., Smith, L. M., LaGasse, L. L., Derauf, C., Grant, P., Shah, R., ... & Della Grotta, S. (2010). Intrauterine growth of infants exposed to prenatal methamphetamine: results from the infant development, environment, and lifestyle study. The Journal of pediatrics, 157(2), 337-339.
16. O'Connor, A., Seeber, C., Harris, E., Hamilton, D., Sachmann, M., & Fisher, C. (2020). Developmental outcomes following prenatal exposure to methamphetamine: A Western Australian perspective. Journal of Paediatrics and Child Health, 56(3), 372-378.
17. Perez, F. A., Blythe, S., Wouldes, T., McNamara, K., Black, K. I., & Oei, J. L. (2021). Prenatal methamphetamine-impact on the mother and child-a review. Addiction (Abingdon, England), 10.1111/add.15509. Advance online publication. **https://doi.org/10.1111/add.15509**
18. Schmidt, U., Capek, C., Birdir, C., Erfurt, C., & Nitzsche, K. (2019). " Crystal" and Pregnancy-The Preliminary Results of a Retrospective Study on the Course of Pregnancy and Delivery of Women Consuming Methamphetamine. Zeitschrift fur Geburtshilfe und Neonatologie, 223(4), 221.
19. Schreiter, J., Vogel, M., Kiep, H., Thome, U., Blaeser, A., Nickel, P., ... & Bernhard, M. K. (2019). The Cognitive, Language and Motor Development of Prenatal Methamphetamine-and Opioid-exposed Children. Klinische Padiatrie, 231(5), 262-268.
20. Shah, R., Diaz, S. D., Arria, A., LaGasse, L. L., Derauf, C., Newman, E., ... & Della Grotta, S. (2012). Prenatal methamphetamine exposure and short-term maternal and infant medical outcomes. American journal of perinatology, 29(5), 391.
21. Western Australian Centre for Evidence Based Nursing & Midwifery (2007). Breastfeeding Guidelines for Substance Using Mothers, Western Australian Centre for Evidence Based Nursing & Midwifery, Perth.
22. Wouldes, T., LaGasse, L., Sheridan, J., & Lester, B. (2004). Maternal methamphetamine use during pregnancy and child outcome: what do we know. NZ Med J, 117(1206), 1-10.
23. Wright, T., Schuetter, R., Tellei, J. & Sauvage, L. (2015). Methamphetamines and Pregnancy Outcomes. Journal of Addiction Medicine, 9(2), pp.111-117.

WHAT ARE CO-OCCURRING CONDITIONS ('COMORBIDITY')?

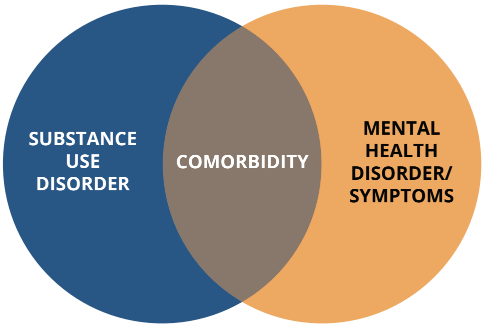

‘Comorbidity’ occurs when a person experiences two or more medical conditions at the same time. There are many different types of comorbidity that people can experience. This factsheet focuses on the type of comorbidity where someone experiences both a substance use disorder (e.g. crystal methamphetamine ‘ice’ dependence) and a mental health disorder (e.g. anxiety, depression, psychosis) at the same time. Another term used for this is ‘co-occurring substance use and mental health disorders’.

To learn more a about the mental health problems that can happen when someone is using ice please visit the mental health effects of ice page.

HOW DO THEY DEVELOP?

There are several reasons why substance use and mental health disorders might occur together. The important thing to remember is that, once established, these conditions often interact in ways that maintain one another. This can make it hard for people to recover if they are not able to access treatment for both problems.

ONE CONDITION MAY DIRECTLY CAUSE THE OTHER

Someone may become dependent on alcohol and/or other drugs (like ice) because they use them to help cope with mental health symptoms.

Alternatively, someone’s use of alcohol and/or other drugs may impair the way their brain functions, leading to poor mental health.

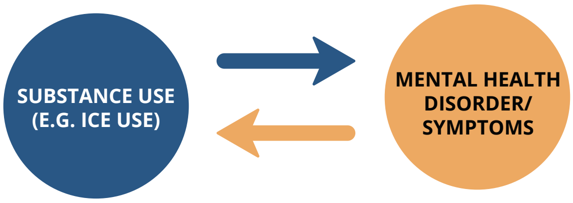

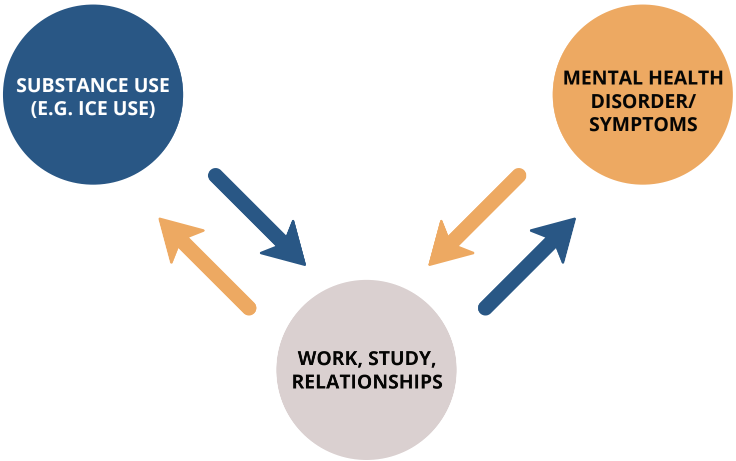

ONE CONDITION MAY INDIRECTLY CAUSE THE OTHER

Poor mental health may cause life difficulties that lead someone to use alcohol and/or other drugs. For example, the experience of mental health problems may limit someone’s ability to study or work. Someone in this position may start using alcohol or drugs to manage the stress of not being able to study or work how they would like to.

In the opposite direction, using alcohol and/or other drugs may limit someone’s ability to study or work. The stress of not being able to study or work how they would like to, may then impair their mental health.

BOTH CONDITIONS MAY BE CAUSED BY SOMETHING ELSE

Sometimes two conditions can be caused by a shared biological, psychological, social or environmental risk factor. A shared risk factor is something about a person or their circumstances that increases their risk of experiencing each of the conditions.

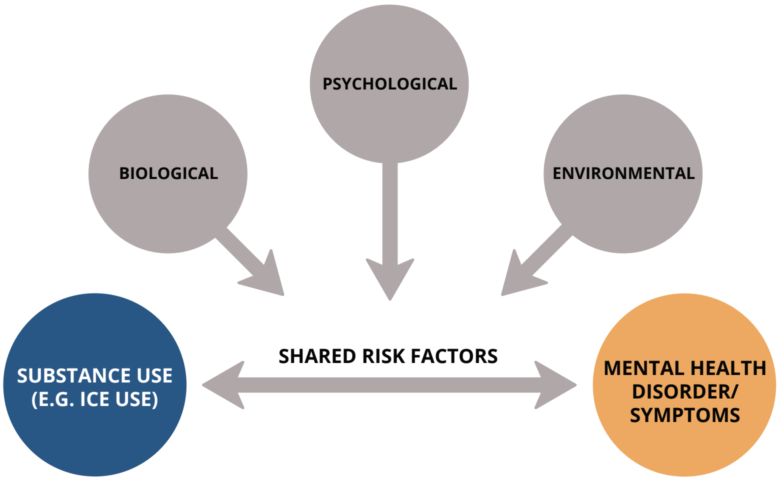

HOW COMMON ARE THEY?

Substance use disorders and mental health disorders are both common in Australia. National population estimates indicate that one in two Australians will develop a substance use, anxiety or mood disorder (e.g. depression) in their lifetime, and one in five meet criteria for a substance use, anxiety or mood disorder annually.

The ***co-occurrence*** of substance use disorders and mental health disorders is also common.

1 in 3

individuals with a substance use disorder also have at least one co-occurring anxiety or mood disorder.

3 in 4

clients in Australian alcohol and other drug treatment services have at least one co-occurring mental health disorder.

WHAT ARE THE HARMS ASSOCIATED WITH CO-OCCURRING CONDITIONS?

People with co-occurring substance use (e.g. ice dependence) and mental health disorders generally experience poorer overall health and wellbeing compared to those who have just one of these conditions. See the figure below for a summary of the harms associated with comorbidity.

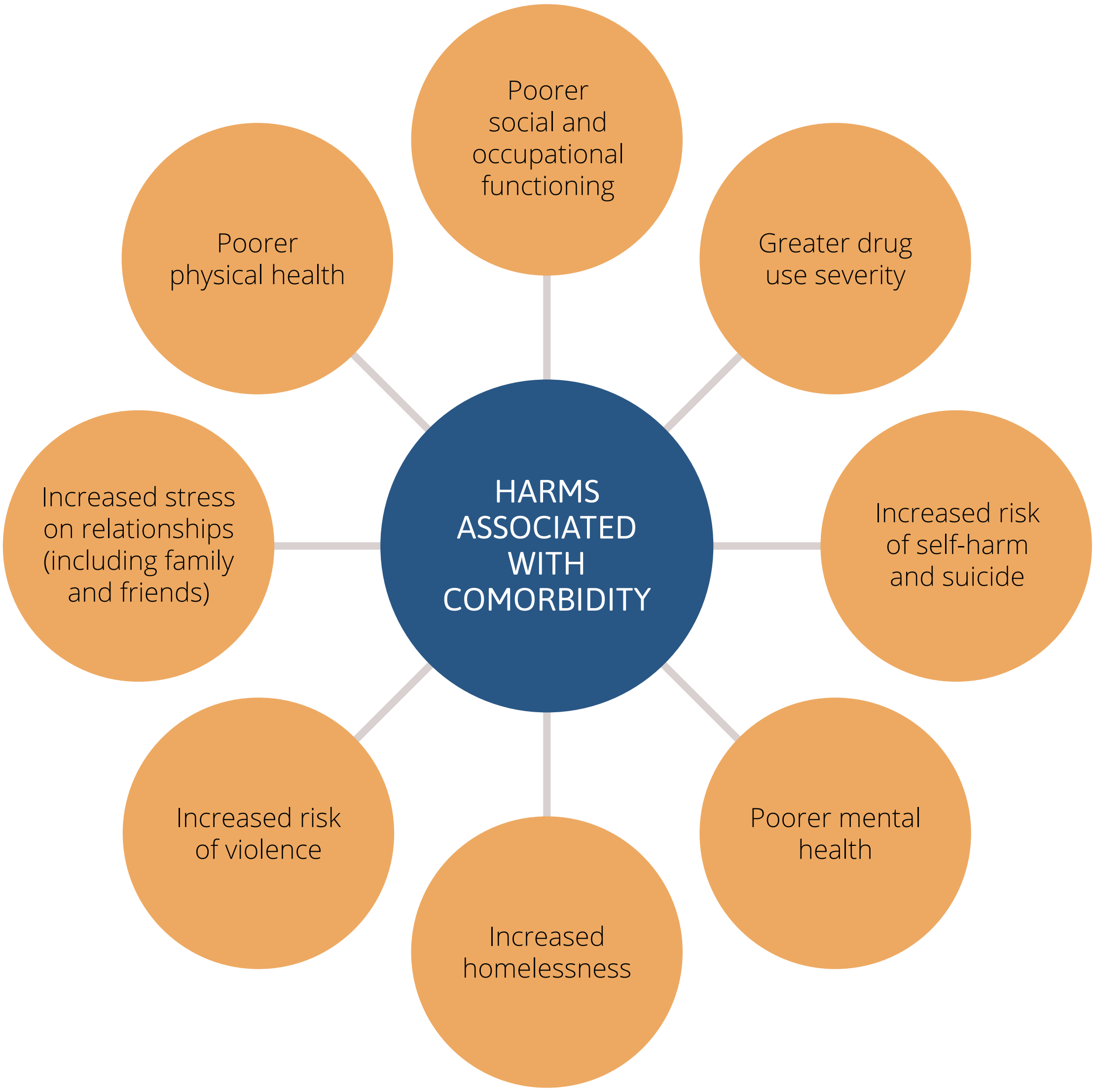

Adapted from the Comorbidity Guidelines. See the key source list below for more information.

DO THEY AFFECT TREATMENT?

Research into whether people who have a co-occurring mental health condition respond differently to alcohol and other drug treatment is mixed.

On balance, the evidence indicates that **having co-occurring conditions is not an insurmountable barrier to treating people with alcohol and other drug use disorders, however some clients with co-occurring mental health disorders may require additional treatment.** People experiencing co-occurring substance use and mental health issues should discuss this with their local doctor, alcohol and other drug counsellor or mental health professional to ensure both areas of concern are addressed in treatment.

For more information about treatment and support services available visit the get help page.

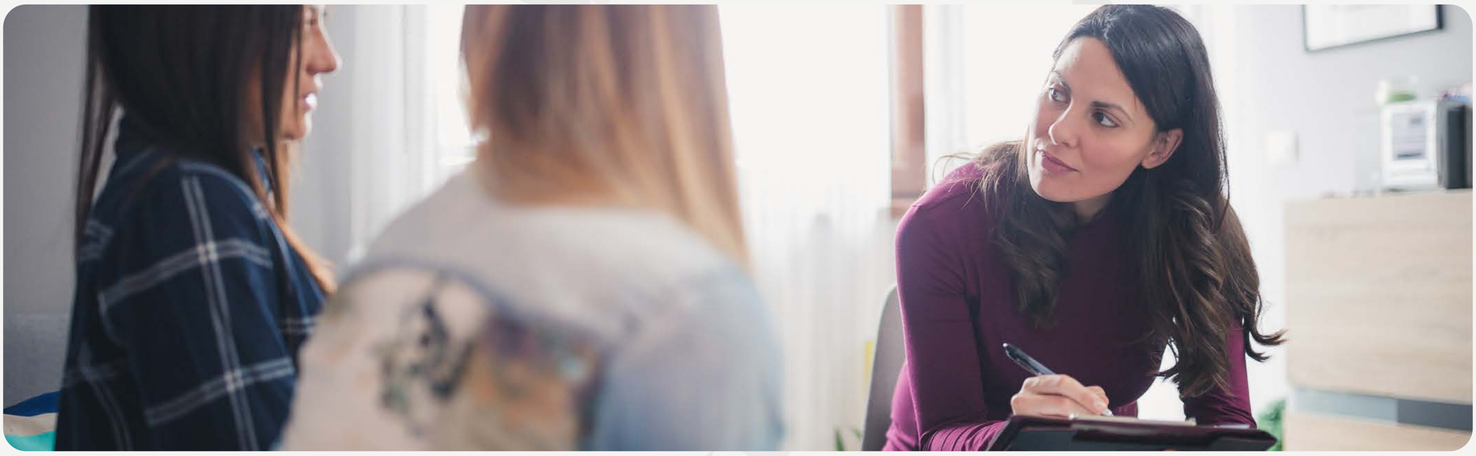

REFERENCES:

Information in this fact sheet has been adapted from the Guidelines on the management of co-occurring alcohol and other drug and mental health conditions in alcohol and other drug treatment settings (3rd edition). The full Guidelines can be accessed **here**. A full list of references is available **here**.

Positive Choices
Drug and Alcohol Information
Get informed, stay smart, stay safe

Factsheet

How to help someone who has taken a drug

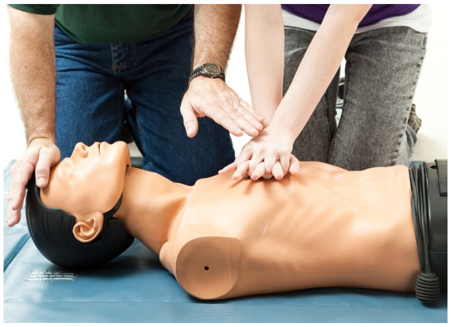

Evidence ratings:

This resource has undergone expert review. See our Help/Q&A section for more details.

Year: Year 7–8, Year 9–10, Year 11–12

Targeted Drugs: Drugs (General)

Tags: first aid, emergency, recovery position, DRS-ABCD, basic life support flow chart, overheating, dehydration, seizures, convulsions, panic attacks, CPR

Origin: Australian

Cost:
Free

Helping someone who has taken a drug

The majority of people never use drugs, but it is important for people to know how to assist someone if a drug-related emergency happens. Drugs are unpredictable; they can affect people in different ways. As there is no quality control for illegal drugs there is no way of knowing their content or strength.

Call 000 for an ambulance immediately if there is even the slightest risk that someone is having an unusual reaction to a drug.
A parent or guardian will only be notified if the person is under the age of 18 and taken to hospital. Police will only be notified if there is a risk to their own personal safety or if someone dies.

Below is a guide to assisting a person in the following situations:

Panic attacks

These can happen due to the increased feelings of paranoia, anxiety, and hallucinations that illegal drugs can bring on. These can be very frightening at the time, but it is important to know that these usually pass with time.

What are some of the signs?

- Sweating and shaking;
- Chest pains and difficulty breathing;
- Increased heart rate;
- Sense of impending death;
- Dizziness, headaches, and light headedness;
- 'Spaced-out' and non-responsiveness.

What to do if someone has a panic attack?

- Calm them down and reassure them that the feeling will pass;
- Take them somewhere cool and quiet away from crowds and bright lights;
- Encourage them to relax and take long, slow, deep breaths;
- If they pass out due to over-breathing, follow the DRS-ABCD life support chart (see below).

Overheating and dehydration

There is a serious risk of overheating and dehydration when people do not maintain their fluids when taking drugs. Stimulants such as MDMA/ecstasy and methamphetamine will increase the body temperature. This can be made worse if taken while drinking alcohol which further dehydrates the body. Those who take stimulants should try to drink half a litre of water every hour, but make sure not to drink too much too quickly.

What are some of the signs?

- Feeling hot, unwell, lethargic, faint, or dizzy;
- Inability to talk properly;
- Headache;
- Vomiting;
- Inability to urinate or urine becoming thick and dark;
- Not sweating even when dancing;
- Fainting, collapsing, or convulsing.

What to do if someone becomes overheated and dehydrated?

- Take them somewhere cool and quiet such as the first aid area or 'chill-out' room;
- Get the person some cold water and get them to sip it slowly;
- Make sure someone stays with them;
- Give them salted foods like crisps or peanuts to replace salts lost through sweating;
- Fan them to cool them down;
- If symptoms persist or get worse seek first aid immediately, call 000, or take them to the nearest emergency department.

Feeling very drowsy

If someone becomes very drowsy from using drugs they could fall asleep and lose consciousness. It is important to keep them awake while waiting for the ambulance.

What to do if someone becomes very drowsy?

- Call an ambulance, but make sure they are not left on their own;
- Keep them awake; make them walk around or make them talk to you;
- Don't give them coffee or try to shock them;
- If they aren't responsive or lose consciousness put them in the recovery position.

Fits or seizures (convulsions)

Large amounts of alcohol and some drugs can cause convulsions, otherwise known as a fit or seizure.

What to do if someone starts convulsing?

- Call an ambulance;
- Clear the area of any nearby harmful objects;
- Loosen any tight clothing;
- Cushion their head;
- It is important not to put anything in their mouth or to try and restrict their movement;
- Once the fit has finished, check their breathing and put them in the recovery position.

A person collapses

If a person collapses it may be necessary to perform cardiopulmonary resuscitation (CPR). CPR can temporarily maintain circulation to the brain to keep it functioning. An easy way to remember the steps involved in this process is to learn the acronym DRS-ABCD (see below).

Doctor's DRS-ABCD: Basic Life Support Flow Chart

D - check for DANGER

- First ensure that your safety is not at risk.
- If your safety is assured and the person is in danger move them out of the dangerous situation.

R - check for RESPONSE

- Ask them their name or to open their eyes.
- If they respond, help to make them comfortable and continue to monitor their response
- If you don't get a response, or they stop responding, send for help

S - SEND for help

- If you receive no response call for an ambulance on ‘000’.
- If you are on your own with the person, first place them in the recovery position and then call ‘000’.
- If you know what drugs the person has taken tell the operator and ambulance officers.
- Ask them their name or to open their eyes.

A - open the AIRWAYS

- If the airway is not clear, place them in the recovery position and open and clear the airway.
- If the airway is clear, leave on back, then tilt the head backwards and lift the chin.

B - check for BREATHING

- Look and feel for chest movements. Listen for breathing from airways.
- If they are breathing, place them in the recovery position and monitor until ambulance arrives.
- If the person is not breathing, place the person on their back, pinch their nose closed, seal your lips over their mouth and give two initial breaths, ensuring that the chest rises with each breath.

C - CPR

- If they are still not breathing, commence CPR until the ambulance services arrive.
- Place one hand on top of the other, palms facing down, over the centre of the chest.
- Compress the chest one third of the depth, 30 times, at a rate of two per second.
- Give 2 breaths for every 30 chest compressions (mouth to mouth can be considered unnecessary).
- Continue CPR until signs of life return, qualified help arrives, or it is impossible to continue (e.g. exhaustion).

D - Attach an automated external DEFIBRILLATOR if available and follow the prompts.

Evidence Base

This factsheet was developed following expert review by researchers at the Matilda Centre for Research in Mental Health and Substance Use at the University of Sydney, the National Drug & Alcohol Research Centre at the University of New South Wales, and the National Drug Research Institute at Curtin University.

See Teacher Booklet, Parent Booklet or Student Booklet for more information.

© 2023 Positive Choices | Built by Netfront

Positive Choices

Drug and Alcohol Information

Get informed, stay smart, stay safe

Drugs a-to-z

Cannabis: Factsheet

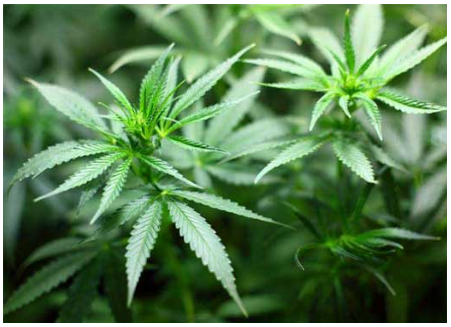

Evidence ratings:

This resource has undergone expert review. See our Help/Q&A section for more details.

Year: Year 9–10, Year 11–12

Targeted Drugs: Cannabis

Tags: pot, hash, marijuana, yarndi, ganja, mull, weed, hash, dope, bush, hydro

Time Allocated: Partial lesson (under 45mins)

Origin: Australian

Cost:

Free

What is Cannabis?

In Australia, cannabis is also known as grass, pot, dope, weed, joints, mull, hydro, yarndi, ganja, bud,or green.

Cannabis is derived from the plant *Cannabis sativa*. The main active ingredient responsible for the 'high' produced by cannabis is called delta-9-tetrahydrocannabinol or THC.

Cannabis is produced in three main forms:

- **Cannabis herb** (also known as marijuana) - the dried flowering tops/buds and leaves of the cannabis plant.
- **Cannabis resin** (also known as hashish) - the resin of the cannabis plant.
- **Cannabis oil** (also known as hash oil) - a thick oil obtained from hashish.

While hashish and hash oil contain more THC than marijuana, they are not widely used in Australia. Cannabis is typically smoked and often mixed with tobacco. It is also sometimes added to food and eaten.

How many young people have tried Cannabis?

According to the 2017 Australian secondary schools' survey, 1 in 12 students (8%) aged 12-17 used cannabis in the past month.

Personal Stories

"The last time I had a joint was one of the worst nights of my life. I felt like I wasn't aware of time and that I was losing my mind. My heart was racing and I thought I was going to have a heart attack. It's difficult to explain but it was a truly terrifying experience that has made me never want to touch weed again."
-Thomas, 15

What are the effects of Cannabis?

If smoked, the effects are often felt quickly as THC is rapidly absorbed into the lungs and can enter the bloodstream within minutes.

The effects of cannabis can be immediate or long term, as listed in the table below.

Immediate	Long-term
Increased appetite	Problems with memory and learning
Feeling relaxed or drowsy	Dependence (see glossary)
Loss of co-ordination	Decreased motivation and concentration
Loss of inhibitions	Increased risk of respiratory diseases
Bloodshot eyes	Paranoia
Dryness of the mouth and throat	Psychosis
Lethargy or tiredness	
'Greening out' (feeling sweaty, dizzy, nauseous, vomiting)	
Anxiety and panic attacks	
Paranoia (feeling extremely suspicious)	
Psychosis (a serious mental illness that causes people to misinterpret or confuse reality)	

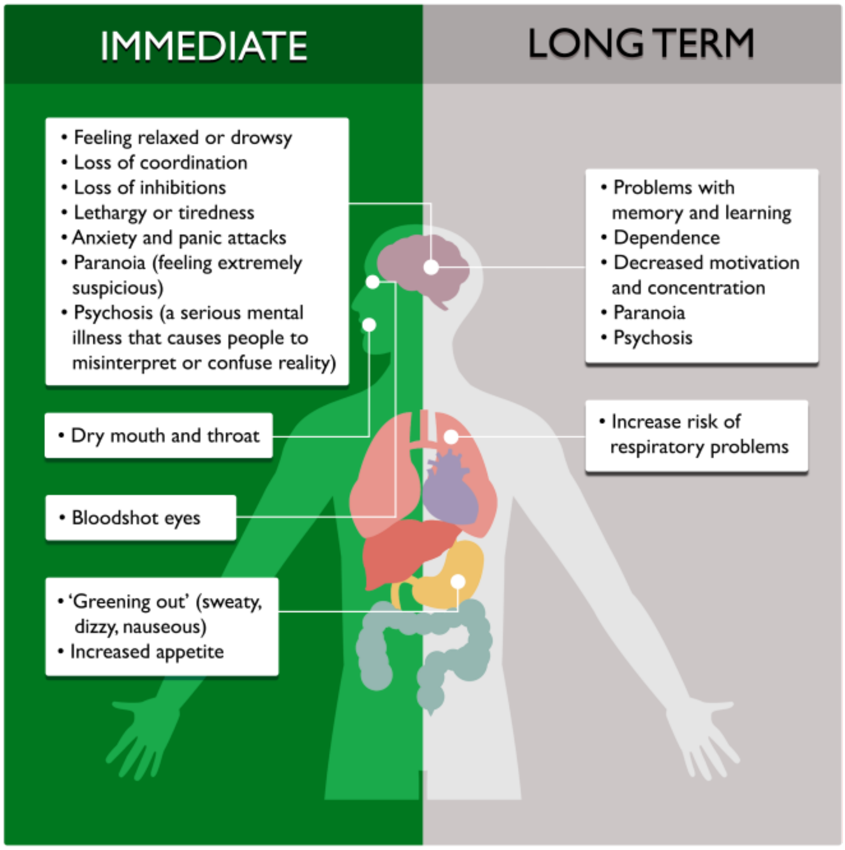

Common Myths

Myth: It is safe to drive after using cannabis.

Using cannabis can increase the likelihood of a car crash by 2-3 times (200-300%) with some studies presenting higher estimates. Cannabis slows down thinking, reflexes and reduces concentration and co-ordination. As a result, cannabis affects the way you do tasks and activities.

Evidence Base

This factsheet was developed following expert review by researchers at the Matilda Centre for Research in Mental Health and Substance Use at the University of Sydney, the National Drug & Alcohol Research Centre at the University of New South Wales, and the National Drug Research Institute at Curtin University.

See Teacher booklet, Parent Booklet or Student Booklet for more information and a list of sources.

- Credit to the Home Office for quotes adapted from Talk to Frank

© 2023 Positive Choices | Built by Netfront
